# Beta-Adrenergic Agonists 

**DOI:** 10.3390/ph3041016

**Published:** 2010-03-30

**Authors:** Giovanni Barisione, Michele Baroffio, Emanuele Crimi, Vito Brusasco

**Affiliations:** 1 Preventive and Occupational Medicine Unit, Respiratory Pathophysiology Laboratory, University Hospital San Martino, Largo R. Benzi, 10 - 16132 Genoa, Italy; 2 Respiratory Pathophysiology Unit, Department of Internal Medicine, University of Genoa, Viale Benedetto XV - 16132 Genoa, Italy; E-Mails: michele.baroffio@unige.it (M.B.); emanuele.crimi@unige.it (E.C.); vito.brusasco@unige.it (V.B.)

**Keywords:** β_2_-adrenoceptors, G-protein-coupled receptor signaling, airway smooth muscle, bronchodilation, desensitization, safety issues

## Abstract

Inhaled β_2_-adrenoceptor (β_2_-AR) agonists are considered essential bronchodilator drugs in the treatment of bronchial asthma, both as symptoms-relievers and, in combination with inhaled corticosteroids, as disease-controllers. In this article, we first review the basic mechanisms by which the β_2_-adrenergic system contributes to the control of airway smooth muscle tone. Then, we go on describing the structural characteristics of β_2_-AR and the molecular basis of G-protein-coupled receptor signaling and mechanisms of its desensitization/ dysfunction. In particular, phosphorylation mediated by protein kinase A and β-adrenergic receptor kinase are examined in detail. Finally, we discuss the pivotal role of inhaled β_2_-AR agonists in the treatment of asthma and the concerns about their safety that have been recently raised.

## 1. Introduction

In the past, β-adrenoceptor (β-AR) agonists have been utilized in many clinical settings. Nowadays, they are considered as first-line medications in the treatment of airway narrowing, the hallmark feature of bronchial asthma and chronic obstructive pulmonary disease (COPD). Epinephrine was the first bronchodilator used at the beginning of the past century. Ephedrine was introduced into Western medicine in 1924, although it had been used in China for thousands of years [1]. The next major advance in this field was the development in the 1940s of isoproterenol [2], a β-AR selective agonist void of α-adrenergic activity. More recently, the development of selective β_2_-AR agonists has made available drugs with reduced cardiovascular effects [3]. Therefore, β_2_-AR agonists represent today a mainstay of the management of COPD [4] and asthma. In the latter condition, they are used both as symptoms-relievers and, in combination with inhaled corticosteroids, as disease-controllers [5,6].

The aim of this article is to review the molecular mechanisms by which β_2_-AR contribute to control the tone of airway smooth muscle (ASM) and their implications for practical use of β_2_-AR agonists in the treatment of asthma.

## 2. Adrenergic Control of ASM Tone

In humans, unlike in other species, there is no evidence that adrenergic nerves directly supply the ASM [7], the only inhibitory nervous system being the non-adrenergic, non-cholinergic one [8,9,10]. The sympathetic system affects ASM tone *via* circulating cathecolamines [7,8,9] acting on the β_2_-AR present on ASM cell membrane and on parasympathetic nerve endings [11,12].

### 2.1. Circulating Catecholamines

Epinephrine, nor-epinephrine, and dopamine are natural circulating compounds, but only the first has significant physiological effects. Following secretion from adrenal medulla and at plasma concentrations ranging from 0.2 to 0.4 nM/L, epinephrine provides a low-level stimulation of specific receptors [13], while spillover of nor-epinephrine from adjacent structures may also exert some minor effects. The action of epinephrine is rapidly (within about 2 min) terminated in various tissues as a consequence of oxidative deamination and methylation processes catalyzed by monoamine oxidase (MAO) and catechol-*O*-methyltransferase (COMT), respectively [14]. 

In asthmatic but not healthy subjects, β-AR antagonists (β-blockers) often elicit bronchoconstriction, which may be interpreted as suggesting a protective effect of β-AR stimulation against excessive airway narrowing. However, no evidence of plasmatic epinephrine was provided during acute exacerbations of asthma or airway narrowing induced by a variety of challenges, including intravenously administered propranolol. Therefore, it does not seem that severe bronchoconstriction and activation of ASM elicit a protective epinephrine release [15].

### 2.2. Neural Interactions

Complex interactions between various components of the autonomic nervous system have long been recognized in various species. This means that changes in the function of one neural pathway may affects other pathways. In humans, ASM tone is mainly maintained by acetylcholine (ACh) released from the parasympathetic nervous system [16]. An early study, based on isometric measurement of tension in bronchial rings, suggested that stimulation of β_2_-AR could inhibit cholinergic neurotransmission [17]. As tyramine-induced release of nor-epinephrine from sympathetic nerves had no effect, this modulation was probably due to circulating epinephrine [17]. Therefore, apart from the relaxing effect on ASM, β_2_-AR may have a modulatory effect on cholinergic neurotransmission [17,18] (*see below*). It has been proposed that an impairment of modulation of ACh release in asthmatic airways may contribute to bronchoconstriction induced by β-blockers [19].

## 3. Adrenergic Receptors

Ahlquist [2] proposed in 1948 that adrenergic receptors, stimulating a variety of physiological responses in various organs, could be classified into two primary types, α and β. Stimulation of the former causes ASM contraction with the following rank of potency: epinephrine > nor-epinephrine > isoproterenol. By contrast, stimulation of β-AR, by the same agonists, relaxes ASM with a different rank of potency, *i.e*., isoproterenol > epinephrine > nor-epinephrine. 

As a result of studies conducted by Lands *et al.* [20], β-AR have been further classified into β_1_- and β_2_-subtypes. The former show an almost equal affinity for epinephrine and nor-epinephrine, the latter are considered to be more sensitive to epinephrine than nor-epinephrine. Autoradiographic mapping has demonstrated that β-AR are widely distributed in the lung and are present in several cell types, including ASM from trachea down to the terminal bronchioles [21]. The presence of β_1_-AR in different species depends on the density of ASM adrenergic supply and the level of bronchial tree [22]. Consistent with the absence of sympathetic innervation to ASM in humans is the autoradiographic evidence of β_2_-AR only in ASM at any airway generation [21]. The amount of β_2_-messenger ribonucleic acid (mRNA) in ASM is high relative to the low receptor density, which may indicate a rapid turnover of β_2_-AR and may account for the relative resistance to the development of tolerance [23].

Apart from ASM relaxation, other effects of β_2_-AR activation have been reported, including increase in ciliary beat-frequency, changes in vascular permeability, decrease in ACh release, and modulation of immune cells function [21,23]. Whether these responses may contribute to the therapeutic efficacy of β_2_-AR agonists in asthma treatment remains unclear. More recently, a third subtype (namely, β_3_-AR) has been demonstrated in isolated canine [24] but not in humans [25] ASM. Its main action seems to be the enhancement of lipolysis in adipose tissue and thermogenesis (“brown” fat) in skeletal muscle [26].

Functional studies have shown that ASM relaxation, at the level of both central and peripheral human airways, is mediated solely by β_2_-AR [27,28]. In asthmatics, selective stimulation of β_1_-AR by prenalterol has no bronchodilator action [29]. Most importantly, β_2_-AR agonists act as functional antagonists and inhibit or reverse contractile responses, irrespective of constrictor stimuli [30,31]. This is a property that is of particular interest in asthma, where several physical or chemical spasmogens are likely to be involved. However, in COPD anticholinergics may produce equivalent or even greater bronchodilation than β_2_-AR agonists because vagal tone is the major reversible element in such patients [32].

## 4. G-Protein-Coupled Receptor Signaling

### 4.1. ASM Relaxation

The β_2_-AR belongs to the ubiquitously expressed 7-transmembrane receptors superfamily, which classically signals through heterotrimeric G-proteins [33,34]. They are commonly referred to as G-protein-coupled receptors because accomplish signal transduction to the interior of the cell *via* interactions with guanine nucleotide regulatory binding proteins [35]. The receptor-coupled G-proteins function as “molecular switches” alternating from an inactive guanosine-diphosphate to an active guanosine-triphosphate (GTP) state, which proceeds to regulate downstream cell processes [35]. Signaling *via* many hormones and neurotransmitters, as well as photons and odors, follows the same basic scheme, *i.e.*, by binding of an extracellular ligand to the receptor, which then interacts with the membrane-bound G-protein. This complex, often referred to as the ternary complex, then acts through the activated G-protein to modulate an effector, such as adenylyl cyclase, phospholipase C, or ion channels [35]. For more details on this argument, we refer the reader to the review by Dohlman [36]. 

**Figure 1:**
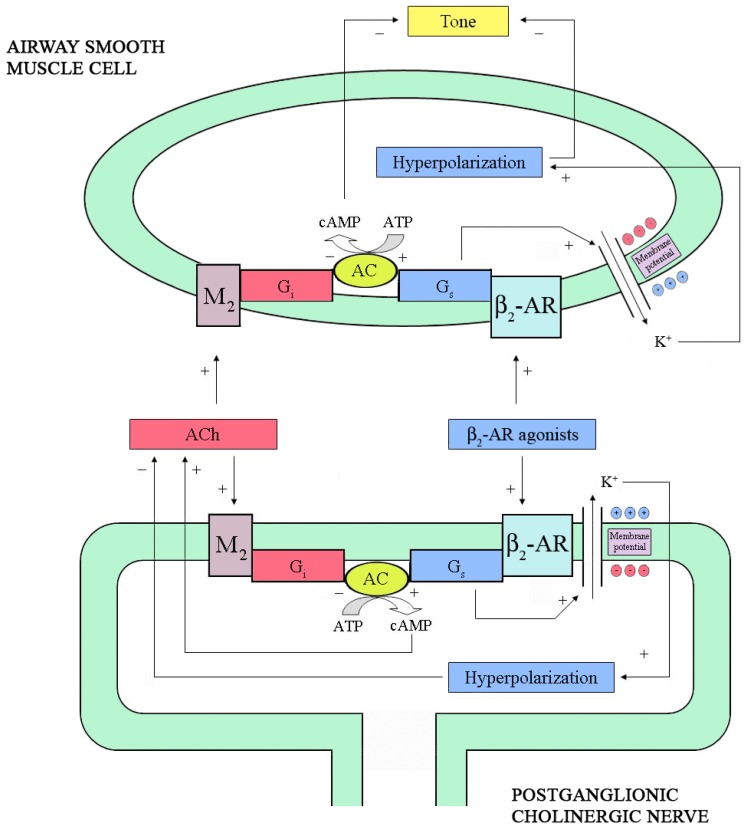
Pre- and post-junctional intracellular mechanisms modulating cholinergic neurotransmission and airway smooth muscle (ASM) cell tone. At pre-junctional level, stimulation of β_2_-adrenoceptor (β_2_-AR) by agonists opens Ca^2+^-activated K^+^ (BKCa) channels leading to cell membrane hyperpolarization and reduction of acetylcholine (ACh) release. By contrast, direct activation of adenylyl cyclase (AC) enhances ACh release. In the ASM cell, stimulation of β_2_-AR as well as direct stimulation of AC, opens BKCa channels determining cell membrane hyperpolarization and relaxation. The ACh released by postganglionic cholinergic nerves binds M_2_-muscarinic receptors expressed both at pre- and post-junctional level, thus inhibiting ACh release and increasing ASM cell tone. cAMP: cyclic 3',5'-adenosine monophosphate; ATP: adenosine trisphosphate; G_s_ and G_i_: stimulatory and inhibitory subunits of the receptor-coupled G-protein, respectively.

Activated β_2_-AR promotes the binding of a heterotrimeric stimulatory G-protein termed G_s_ (consisting of α, β, and γ subunits) to the receptors [37]. Upon binding of GTP, the G_s_-protein subunits disassociate into G_α_ and G_βγ_. Traditionally, G_α _was considered to be the subunit that stimulates adenylyl cyclase (AC), but G_βγ_ can also activate certain pathways [37]. The resulting increase in intracellular cyclic 3',5'-adenosine monophosphate (cAMP) concentrations activates protein kinase (PKA), which phosphorylates several proteins causing relaxation [38]. The intrinsic GTPase activity of G_α _subsequently terminates the process, with re-association of heterotrimeric protein [37]. In bronchial smooth muscle, PKA inhibits both myosin-light-chain kinase [39] and phosphoinositide hydrolysis [40]. Moreover, it promotes Ca^2+^/Na^+ ^exchange [41], thus resulting in a decrease of intracellular [Ca^2+^], and stimulates Na^+^/K^+^ ATPase [42]. However, these effects are only observed at relatively high concentrations of β_2_-AR agonists, when maximal relaxation responses have been exceeded. In the 1990’s, Jones *et al.* [43,44] showed that two potent inhibitors of the opening of the large-conductance Ca^2+^-activated K^+^ (BKCa) channels, *i.e.*, charybdotoxin and iberiotoxin, may inhibit the bronchodilator responses to β_2_-AR agonists and to other agents that can increase cAMP. These effects have been subsequently confirmed by other studies [45,46] and are observed at low concentrations of β_2_-AR agonists in isolated human airways [47]. This suggest that β_2_-AR may activate BKCa channels in ASM directly *via* the α-subunit of G_s_ [48] (Figure 1).

Studies, based on direct measurement of ACh release from guinea pig [49,50] and horse [51] trachealis, have shown that stimulation of β_2_-AR enhances cholinergic neurotransmission in the absence, but not in the presence, of epithelium [52]. Consistent with these results, direct activation of the β-AR-coupled G_s_ subunit by cholera toxin, which increases the activity of AC [33], caused an increase of ACh release in epithelium-denuded guinea pig trachealis [49].

Moreover, direct stimulation of AC by forskolin [53] or incubation with a cAMP analog, increased ACh release [49]. In ASM cells, however, stimulation of G_s_ protein directly opens BKCa channels [48], which has been found to decrease ACh release in guinea pig trachealis [54]. By simultaneously measuring electrically induced ACh release and force development, Brichetto *et al.* [55] from our laboratory have recently shown that β_2_-AR agonists attenuates cholinergic neurotransmission in bovine trachealis by a mechanism involving BKCa channels. Therefore, it can be hypothesized that β_2_-AR agonists have the potential to increase or decrease ACh release depending on whether they mainly act through AC or BKCa channels. 

Previous studies have shown that β_2_-AR can activate PKA-independent mechanisms to elicit functional responses [45,46]. Freund-Michel *et al.* [56], have recently found that the anti-tussive properties of terbutaline were abolished by an inhibitor of protein kinase G (PKG) present in vagal sensory fibres but were unaffected by a PKA inhibitor. As the level of cyclic 3’,5’-guanosine monophosphate was not elevated in the vagus nerve, these data suggest that a cAMP-dependent cross-activation of PKG and, subsequently, the opening of the BKCa channels may account for the anti-tussive properties of β_2_-AR agonists. If bronchodilator and anti-tussive activity of β_2_-AR agonists is mediated by different signaling pathways, then some discrepancies, reported in the clinical literature [57,58,59,60] regarding their anti-tussive and bronchodilator efficacies, could be explained.

### 4.2. Anti-Inflammatory Effects

It has been proposed that actions other than ASM relaxation may play a role in the protective effect of long-acting β_2_-AR agonists against allergen-induced late asthmatic reactions [61]. In diseases such as asthma, ASM cells play a synthetic role by secreting inflammatory mediators [62]. In other words, ASM may produce multiple inflammatory mediators (prostanoids, cyto- and chemokines), thus being able to perpetuate and amplify the inflammatory process within the airway wall. Evidence is rapidly accumulating that the asthmatic ASM is abnormal, in that it proliferates faster [63,64], produces more chemokines and cytokines as well as a different profile of extracellular matrix proteins than in its non-asthmatic counterpart [62]. These abnormalities may stem from altered calcium homeostasis leading to increased mitochondrial biogenesis and/or decreased levels of key transcription factors, such as CCAAT enhancer binding protein-α [65]. Recent data indicate that both short- and long-acting β_2_-AR agonists are able to reduce the expression of surface intercellular adhesion molecule-1 and the release of granulocyte-macrophage colony-stimulating factor evoked by interleukin-1β in ASM [66]. Interestingly, the repression of these inflammatory indexes was prevented by adenoviral over-expression of PKI-α, a highly selective PKA inhibitor. These data indicate a PKA-dependent mechanism of repression and suggest that agents that elevate intracellular cAMP, and thereby activate PKA, may have an anti-inflammatory effect in ASM cells [66]. 

## 5. β_2_-AR Dysfunction

When a cell or tissue is first exposed to a β_2_-AR agonist, there is a brisk initial response in the production of cAMP, followed by a decline to nearly basal level despite the continued presence of the drug. The cell becomes “desensitized” or “tolerant” to further stimulation. Desensitization depends on both the concentration of β_2_-AR agonist and time of exposure. In any cell type, the process of desensitization occurs through an acute and a chronic phase [67].

Over the years, there has been some confusion regarding the terminology used to describe receptors dysfunction. More precisely, *desensitization* is defined as a warning of a functional response despite the continuous presence of a stimulus of constant intensity [37]. Typically, a decrease in receptor function over time during exposure of cells or tissues to agonist is one form of desensitization. For β_2_-AR, this would entail measurements of cAMP or AC activities. In intact animal models and in clinical setting, agonist-promoted desensitization is referred to as “tachyphylaxis” or “refractoriness” [37]. 

There is both *in vivo* and *in vitro* evidence that β_2_-AR are less responsive in asthmatic than healthy individuals [68,69]. It is not completely clear, however, whether this decreased responsiveness is the cause of asthma [70] or a byproduct of therapy with β_2_-AR agonists [68]. Trian *et al.* [71] have recently shown that epithelial infection with rhinovirus reduces β_2_-AR function in ASM. This may explain the clinical observation of β_2_-AR dysfunction during virus-induced asthma exacerbations. Some authors reported that the inhibitory effects of interleukin-1β on the isoproterenol-induced bronchodilation are abolished by COX-2 inhibitors [72]. Thus, it appears more likely that β_2_-AR hyporesponsiveness is a consequence of the disease process rather than its cause. 

### 5.1. Desensitization by Phosphorylation

**Figure 2:**
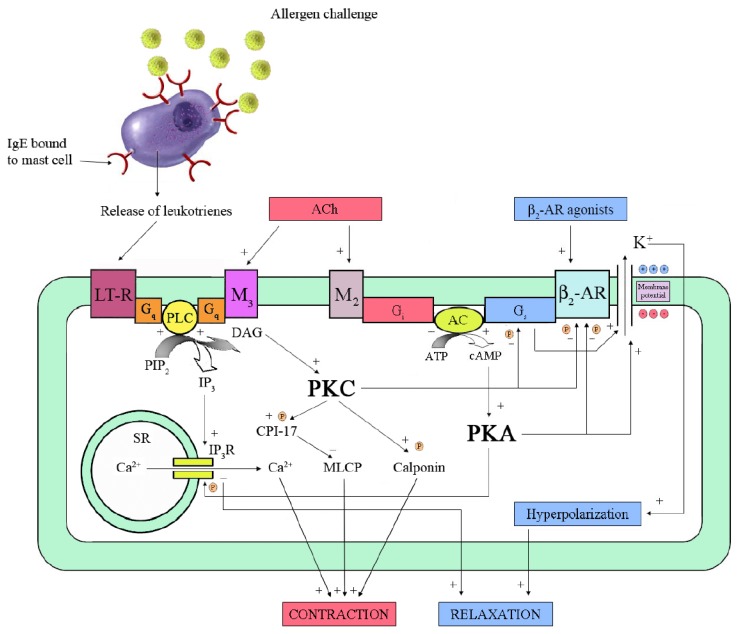
Mechanisms of relaxation and β_2_-adrenoceptor (β_2_-AR) desensitization in airway smooth muscle (ASM). The binding of a specific agonist to β_2_-AR stimulates the receptor-coupled G_s_-protein, which activates adenylyl cyclase (AC). The resulting increase of cyclic 3',5'-adenosine monophosphate (cAMP) activates protein kinase A (PKA), which phosphorylates inositol 1,4,5-trisphosphate receptor (IP_3_R) of sarcoplasmic reticulum (SR) and opens Ca^2+^-activated K^+^ (BKCa) channels, thus leading to relaxation. However, activated PKA phosphorylates β_2_-AR, uncoupling it from G_s_-protein. Exposure of sensitized mast-cells to allergen causes release of leukotrienes (LT). Their interaction with the specific receptor, *i.e.*, LT-R, activates a G_q_-protein, which increases phospholipase C (PLC) activity. PLC catalyzes the hydrolysis of phosphatidylinositol 4,5-bisphosphate (PIP_2_), which produces IP_3_ and diacylglycerol (DAG). IP_3_ leads to contraction by increasing release of Ca^2+^ from SR while DAG activates protein kinase C (PKC). The latter phosphorylates several substrates like calponin and CPI-17, which is an inhibitor of myosin-light-chain phosphatase (MLCP). In addition, PKC phosphorylates both β_2_-AR and G_s_-protein. A similar cascade of events seems to occur in response to acetylcholine (ACh) released by cholinergic nerves through M_3_-muscarinic receptors. In addition, activation of M_2_-muscarinic receptors inhibits AC, thus decreasing cAMP level and PKA activity. ATP: adenosine trisphosphate;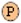: phosphorylation sites.

The covalent modification of β-AR represents the earliest event in the desensitization process [37]. Over a time frame of less than 30 min of exposure to an agonist *in vitro*, phosphorylation of β-AR takes place by PKA and β-AR kinase (βARK). The latter belongs to G-protein-coupled receptor kinases family (GRK) [73]. Both kinases cause a complete or partial loss of functional coupling between receptor and G_s_ subunit by phosphorylating specific sites [67]. The uncoupling between receptor and G_s_ subunit may be a mechanism of allergen-induced β_2_-AR dysfunction observed in human isolated passively sensitized bronchi [73] (Figure 2).

PKA is a cAMP-dependent enzyme which is activated by low concentrations of β_2_-AR agonist, such as circulating catecholamines or drugs. Any process leading to an increase in intracellular concentrations of cAMP sufficient to activate PKA will result in phosphorylation of β_2_-AR, even if unoccupied by agonist [37]. This underlies one of the principal mechanisms of *heterologous desensitization* of the β_2_-AR, so termed because dysfunction can be evoked by processes distinct from activation of the receptor as, for instance, those elicited by inflammatory mediators [67]. Data from our laboratory showed, in passively sensitized isolated human bronchial rings, that allergen challenge caused β_2_-AR dysfunction without involving cytokines [75] or mechanisms distal to cAMP formation [76]. Moreover, this dysfunction was prevented by a LTD_4_-receptor antagonist or a cell membrane stabilizer, but not by cetirizine or indomethacin, suggesting a central role for leukotrienes released from resident inflammatory cells [77]. The involvement of PKC in this process was subsequently shown on both cultured ASM cell and bronchial rings [78]. 

If cysteinyl-leukotrienes, released from resident or circulating inflammatory cells or even from the ASM itself, are responsible for β_2_-AR desensitization in asthma, then the concurrent administration of cysteinyl-LT_1_-R antagonists might represent a useful strategy to improve the response to β_2_-AR agonists in this disease. On the other hand, *homologous desensitization* is mediated by βARK. It describes processes that are specific to an attenuation of function of activated, *i.e.*, occupied, receptor [79]. βARK requires high (μM/mL) concentrations of the agonist [73]. Other data suggest that a cofactor is required for βARK function. A protein, inhibiting the signaling function of βARK-phosphorylated receptors by more than 75%, was found [80] and termed β-arrestin [81]. It is now recognized that β-arrestins act as versatile adapters controlling receptor signaling, desensitization, and trafficking. Most endogenous receptors appear to signal in a balanced fashion using both β-arrestin and G-protein-mediated pathways. Some 7-transmembrane receptors are thought to be non-signaling "decoys" because of their inability to activate typical G-protein signaling pathways. It has been proposed that these receptors act to scavenge ligands or function as co-receptors [82]. 

Nino *et al.* [83] have recently examined the regulation and role of phosphodiesterase-4 (PDE_4_) activity in mediating the pro-asthmatic changes in ASM responsiveness that accompany its prolonged homologous desensitization. Their findings provide new evidence that prolonged exposure of ASM to β_2_-AR agonists results in an upregulated expression of PDE_4_D5 isoform due to PKA-dependent activation of G_i_ protein signaling. This is consistent with a potential switch in coupling of the β_2_-AR from G_s_ to G_i _resulting in G_βγ_-subunit-mediated activation of ERK1/2 which, in turn, leads to a phosphorylating cascade of transcription factors. 

During exposure to low levels of agonist, few β_2_-AR on the cell are occupied [67]. Such low (≤10 nM/mL) concentrations of agonist are present in systemic circulation during exercise-induced activation of the sympathoadrenal system and in stress, such as under haemodynamic instability or respiratory insufficiency. Similar concentrations are also obtained during systemic administration of agonist, such as terbutaline, for the treatment of asthma and various catecholamines for the treatment of hypotension and heart failure [67]. Although only a β_2_-AR fraction is occupied, this is sufficient to increase intracellular cAMP, resulting in activation of PKA and phosphorylation of all β_2_-AR on the cell. Under these conditions, only the agonist-occupied receptors are phosphorylated by βARK, which contributes minimally to the process under these circumstances. Other G-protein-coupled receptors with PKA sites may also be phosphorylated, resulting in desensitization by β_2_-AR agonists [37].

During exposure to high concentrations of β_2_-AR agonists, as occurring in bronchodilator treatment of asthmatic patients, the majority of β_2_-AR on the cell may be occupied [67]. Under these circumstances, PKA is activated, as with low agonist exposure, and all β_2_-AR are phosphorylated by this kinase. In addition, all occupied receptors are phosphorylated by βARK. Such phosphorylated receptor binds β-arrestin with subsequent partial uncoupling from G_s_ [37]. 

Phosphorylation by PKA and GRKs leads to desensitization of β_2_-AR signaling, and is thought to be a mechanism involved with cell and organ homeostasis and tolerance to agonists. However, there is little direct evidence that these events are relevant to β_2_-AR physiological function, such as ASM relaxation. Recently, Wang *et al.* [84] have shed some light on the matter by using a transgenic mouse model expressing the human wild-type and mutated β_2_-AR lacking PKA and/or GRK phosphorylation sites on ASM. They showed that, at low receptor occupancy, β_2_-PKA(-) had enhanced agonist-promoted relaxation, while β_2_-GRK(-) airways were unaffected. In contrast, at saturating agonist concentrations, the greatest relaxation enhancement was with β_2_-GRK(-), with no evidence for additivity when PKA sites were also removed. For the full range of responses, the β_2_-PKA(-)/GRK(-) airways had the greatest relaxation efficiency, indicating a graded effect of GRKs as agonist concentration increased. Thus, these two mechanisms impact a relevant β_2_-AR physiologic function, by acting as attenuators of the acute response, and represent specific interfaces where adjunct therapy or biased ligands may improve β_2_-AR agonist treatment of obstructive lung disease. 

### 5.2. Desensitization by Sequestration

After about 30 min of agonist exposure, cell surface β_2_-AR undergo a process leading to internalization or sequestration of the receptor to a sub-cellular compartment. The proportion of receptors at the surface that undergo sequestration appears to be highly cell-type dependent. In cells where there is little receptor reserve and sequestration results in ~60% loss of cell surface receptor, desensitization by this mechanism would be expected, as shown in lymphocytes [37]. Recent data indicate that the β_2_-AR of human pro-inflammatory cells is exquisitely sensitive to desensitization, whereas β_2_-AR-mediated relaxation of human ASM is relatively resistant. An explanation for this discrepancy is that a large β_2_-AR reserve exists in ASM cells for sympathomimetic bronchodilators, which protects against desensitization [85]. After agonist removal, sequestered receptors, which have not been shuttled to a degradation pathway, can recycle to the cell surface, with a time course that is about the same as for initiation of the process. Some data suggest that the pool of sequestered β_2_-AR undergoes phosphatases-mediated dephosphorylation [86].

### 5.3. Desensitization by Down-Regulation

After 3–6 h of exposure to agonist, a net loss of receptors occurs independent of compartment [37]. This is termed *down-regulation* and reaches a steady state at 18–24 h. The process can result in an loss of as much as 90% of receptors and is presumed to be a major component in long-term agonist-promoted desensitization of β_2_-AR [37]. The mechanisms responsible are both a decrease in receptor production and an increase in receptor protein degradation. The decrease in production of new receptors in the presence of agonist is primarily due to a loss of β_2_-AR mRNA stability [87], although a change in the rate of transcription may also play a role [88]. Destabilization of β_2_-AR mRNA appears to be due to cAMP-promoted binding of a protein to a destabilization sequence (AUUUA) in the 3’ non-coding region of the gene [89]. The second mechanism of down-regulation may be related to the degradation of sequestered β_2_-AR pool. One study suggests that PKA-mediated phosphorylation is required for full agonist-promoted down-regulation [90]. Also, tyrosine residues in the cytoplasmic tail have been implicated as necessary for agonist-promoted down-regulation [91].

### 5.4. The β-AR Paradox

Long-acting β_2_-AR agonists have been introduced as a therapeutic option in asthma [5,6] to improve symptom control. However, there is growing concern about whether control of disease may deteriorate with the regular use of these agents. Several studies have shown a temporary increase in airway responsiveness to histamine upon withdrawal of inhaled β_2_-AR agonists [92,93,94]. In an 8-week study, Cheung *et al.* [95] showed that the bronchodilator effect of salmeterol was maintained, whereas its protective effect against methacholine (MCh)-induced bronchoconstriction was significantly attenuated. This dissociation between bronchodilator and bronchoprotective effect of long-acting β_2_-AR agonists was further explored in mice with ablated β-AR genes (β-AR^-/-^) and transgenic mice overexpressing β_2_-AR (β_2_-AR-OE) [96]. Unexpectedly, β-AR^-^^/-^ mice had markedly decreased bronchoconstrictive responses to MCh and other G_q_-coupled receptor agonists such as thromboxane-A_2_ and 5-hydroxytryptamine (5-HT). In contrast, β_2_-AR-OE mice had enhanced constrictive responses. Inositol phosphate accumulation by G_q_-coupled M_3_-muscarinic, thromboxane-A_2_, and 5-HT_2_ receptors was desensitized in ASM cells from β-AR^-/-^ mice and sensitized in cells from β_2_-AR-OE mice. Thus, β-AR seem to regulate antithetically constrictive signals, affecting bronchomotor tone/reactivity by additional effects other than direct bronchodilation. Studies of signaling elements in these pathways identified the nodal point of this cross-talk in phospholipase C-β1, whose expression was altered by β-AR in a direction and magnitude consistent with the physiologic and cellular responses. These results establish a possible mechanism of *the β-AR paradox* and identify a potential asthma modifier gene (phospholipase C-β1), which may also be a therapeutic target in asthma when chronic β-AR agonists are required [96]. An alternative explanation is that regular use of β_2_-AR agonists may negatively impact the balance of factors contributing to airway inflammation and remodeling in asthma [97].

Two elegant studies [98,99], in ovalbumin-driven murine model of asthma, have shown that β-AR agonists and antagonist, with inverse agonist properties, may exert reciprocating effects on cellular signaling dependent on duration of administration. In particular, acute dosing with β-blockers produced bronchoconstriction, whereas the chronic administration of the same agents did exert a protective effect against MCh-induced airway narrowing [98]. Surprisingly, the bronchoprotective effect of β-blockers was associated with reduced inflammation and mucous metaplasia, up-regulation of β_2_-AR, and reduced expression of various spasmogenic proteins [99]. These findings in mice led to the first proof-of-concept open-label study in ten patients with mild steroid-naïve asthma who were given incremental doses of nadolol for nine weeks [100]. In eight out of the 10 subjects tested, chronic treatment with this β-blocker produced a significant, dose-dependent, decrease in airway responsiveness to MCh. However, these findings warrant further testing in future by means of larger trials [101,102].

### 5.5. Reversal of Desensitization

As detailed by Davies and Lefkowitz [103], corticosteroids have been shown to increase β_2_-AR synthesis, reverse down-regulation, and improve coupling, the last by partially restoring the fraction of receptors in the high-affinity state. A study from our laboratory indicates that short-term (3 h) exposure to beclomethasone dipropionate ablated β_2_-AR dysfunction induced by allergen challenge in passively sensitized human bronchi. This effect was mediated by an increase in the activity of the α-subunit of G_s_ protein, suggesting an action independent of gene transcription [104]. 

## 6. Genetic Polymorphisms of the β_2_-AR

A series of studies revealed that the gene encoding for β_2_-AR is polymorphic within the human population [105,106]. Such polymorphisms may be responsible for heterogeneity in the response to β_2_-AR agonists and/or receptor dysfunction in asthma [107]. For example, the B16 Arg/Arg homozygosity is associated with more frequent exacerbations during chronic daily dosing of salbutamol [108] or salmeterol [109]. In contrast, it seems that there is no genotypic risk for exacerbations in patients using inhaled β_2_-AR agonists less than once a day [109]. In asthma patients with B16 Arg/Arg and B16 Gly/Gly genotypes, combination treatment with salmeterol and inhaled corticosteroid improved airway function when compared with inhaled corticosteroid therapy alone. These findings suggest that patients should continue to be treated with long-acting β_2_-AR agonists plus moderate-dose inhaled corticosteroids irrespective of B16 genotype [110]. 

β_2_-AR function undergoes desensitization during persistent agonist exposure because of receptor phosphorylation by GRKs, which was found to be highly expressed in ASM [111]. The coding region is polymorphic at codon 41, where glutamine (Gln) can be substituted by leucine (Leu) (minor allele), but almost exclusively in those of African descent. So, GRK5-Leu41 represents a gain-of-function polymorphism that evokes enhanced loss-of-function of β_2_-AR during persistent agonist exposure, thus possibly contributing to β_2_-AR agonist variability in asthma treatment of African-Americans [111].

If one considers β_2_-AR expression as being constantly regulated by systemic catecholamines or continuosly administered β_2_-AR agonists, then the glutamic (Glu)27 allele may be expressed to a higher level in the lung, as it is refractory to down-regulation. More than a decade ago, a study by Hall *et al.* [112] addressed this point and found that asthmatics with the Glu27 polymorphism had approximately fourfold lower airway responsiveness to MCh as compared to those with Gln27 (wild type). Recently, a novel gene (arginine1) associated with acute response to inhaled β_2_-AR agonists in both children and adults with asthma was identified [113].

## 7. Role of β_2_-AR Agonists in the Treatment of Obstructive Lung Diseases

### 7.1. Symptom-Relief

#### 7.1.1. Asthma

Short-acting β_2_-AR agonists administered by inhalation are the most effective therapy for rapid reversal of airflow obstruction and prompt relief of asthmatic symptoms. The most widely used are salbutamol (commonly known as albuterol in the US) and terbutaline; their action occurs in 5 min or less, peaks after 30 to 60 min and lasts 4 to 6 h [114]. With regular use of a bronchodilator (four or more times daily), the potency (as measured by the increase in one second forced expiratory volume, FEV_1_) does not decline, but the duration of action is slightly shortened [115]. A regular schedule of administration four times a day does not improve outcomes as compared with as-needed administration [116]. Moreover, in patients with certain genotypic variants of the β_2_-AR it may have a deleterious effect [117,118]. So, the short-acting β_2_-AR agonists are recommended for use on request to relieve symptoms or before anticipated exposure to known asthmatic triggers, especially exercise. In mild-to-moderate asthmatic patients, a cumulative bronchodilator effect of salbutamol can be seen up to inhaled doses ranging from 700 to 1,500 µg [119]. Dose-dependent sympathomimetic-type side effects, including tremor, anxiety, heart pounding, and tachycardia (but not hypertension), are common, and a small dose-dependent decrease in serum potassium and magnesium levels is detectable. However, at the usual dose, adverse effects are uncommon [14].

The decision about which of the various short-acting β_2_-AR agonists to use is based largely on cost and patient's or physician's preference. Salbutamol has been made available in a metered-dose inhaler free of chlorofluorocarbons (CFCs), and CFC-containing salbutamol inhalers were taken off the market on 31 December 2008. Like CFCs, the alternative propellant, hydrofluoroalkane (HFA), is inert in the human airway [120]. The HFA inhalers are equipotent to the CFC-propelled inhalers [121] and can be used with valved holding chambers (spacers) in patients with poor inhalational technique. They provide bronchodilation comparable to nebulized salbutamol when a sufficient number of puffs is administered and inhalational technique is good [122]. Levalbuterol, the purified D-rotatory isomer of salbutamol, was developed on the purpose to eliminate side effects, which some argue are limited to the S-rotatory isomer [123]. However, when delivered by metered-dose inhaler, levalbuterol has an efficacy and side-effect profile that is indistinguishable from that of the racemic mixture of molecules in salbutamol [124].

The inhaled long-acting β_2_-AR agonists salmeterol and formoterol have a bronchodilator potency similar to that of their short-acting counterpart. Features distinguishing the two long-acting β_2_-AR agonists are both practical and theoretical. The onset of action of formoterol occurs within 5 min, a period similar to that for short-acting β_2_-AR agonists, whereas salmeterol has a slower onset of action (15 to 20 min) [14]. For this reason, a combination formoterol-corticosteroid inhaler is recommended both for quick relief of asthmatic symptoms and, when used regularly, for long-term control [125]. Formoterol is a full agonist in its action at the β_2_-AR, whereas salmeterol is a partial agonist (and partial antagonist) [14]. The implication of this pharmacologic distinction, particularly as it might apply to the risk of fatal asthmatic attacks, is uncertain. Both salmeterol and formoterol display a sustained activity (>12 h), and because of their higher degree of β_2_-AR specificity, have fewer side effects than short-acting β_2_-AR agonists [126]. As much as with short-acting β_2_-AR agonists, regular use of long-acting β_2_-AR agonists results in only mild tachyphylaxis to the maximal bronchodilator effect and the duration of action [126]. In contrast, the bronchoprotective effect of long-acting β_2_-AR agonists (*i.e*., their inhibition of exercise-induced bronchoconstriction) rapidly wanes with regular use [127], a contrary pharmacologic effect that has not been fully explained. With rare exceptions [128], the quick symptom relief provided by short-acting β_2_-AR agonists is not impeded by regular use of long-acting β_2_-AR agonists [129].

#### 7.1.2. COPD

In COPD, formoterol and salmeterol are more effective bronchodilators than their short-acting counterpart [130] and have an add-on effect to anticholinergics [131]. Both drugs improve FEV_1_, reduce symptoms and use of rescue medication, and improve exercise capacity and health status [132]. There is also evidence that formoterol may be used as a rescue inhaler without significant unwanted effects [133]. Though tolerance in the long-term use of long-acting β_2_-AR agonists does not appear to be a problem in clinical practice, concerns have been raised about the side effects, particularly cardiac dysrhythmias, in susceptible elderly populations [132]. Nevertheless, salmeterol and formoterol at usual doses (100 µg/day and 24 µg/day, respectively) are effective and safe in treating patients with COPD [134]. Higher doses may cause more untoward effects, although serious adverse events are very uncommon [132].

### 7.2. Disease-Control

The fact that long-acting β_2_-AR agonists afford sustained improvement in lung function may tempt clinicians to use them as a long-term controller medication, without concomitant use of anti-inflammatory treatment. However, this strategy results in unsuppressed airway inflammation and an unacceptably high rate of asthmatic exacerbations [135,136,137,138,139]. For these reasons, the use of long acting β_2_-AR agonists as single treatment for asthma control is not recommended and several formulations combining β_2_-AR agonist with an inhaled corticosteroid in a single device have been made available. Combinations of salmeterol with inhaled corticosteroid are generally used for regular treatment only, whereas those of salbutamol or formoterol have been suggested for both as needed and regular treatment [140].

## 8. The Great β_2_-AR Agonists Controversy

### 8.1. Short-Acting β_2_-AR Agonists

Epinephrine was the first synthetic β-agonist bronchodilator to be used clinically and was introduced in the early 1900’s [141]. As systemic side effects due to potent α- and non-selective β_2_-AR stimulatory effects were reduced but not eliminated by aerosol inhalation, its widespread use for the treatment of asthma was abandoned. Moreover, up to a five-fold increase in mortality among users of inhaled epinephrine was reported in 1948 [142].

Isoproterenol was introduced in 1940 into medicine as short-acting β_2_-AR agonist. It gradually replaced epinephrine because of its more potent β-stimulant properties and its virtual absence of α-agonist activity [143]. However, this epinephrine derivative possesses the same major disadvantage of its parent compound, namely, a relatively short duration of action (due to rapid metabolism mainly by COMT and, to a lesser extent, by MAO) and undesirable cardiac side effects and mortality [143]. 

The most-often-cited examples of the possible relationship between short-acting β_2_-AR agonists and mortality in asthma were the startling rise in asthma deaths in several countries, including the UK, Australia and New Zealand during the early 1960s [144] and in New Zealand in the late 1970s [145,146]. In investigating the earlier epidemic in the UK, Speizer *et al.* [144,147] found that these deaths were most often sudden, unexpected and closely correlated with the increased use of pressurized metered dose inhalers. In the late 1960s, following recognition of the potential danger from the overuse of β_2_-AR agonists and restrictions on their over-the-counter sale, the usage of inhaled short-acting β_2_-AR agonists declined. Simultaneously, the asthma death rate fell sharply, suggesting that the previously noted relationship might have been a causal one. It is noteworthy, however, that this decline in the asthma death rate occurred concomitantly with a greater reliance on corticosteroid therapy, suggesting that the latter might also have contributed to the drop in mortality due to asthma [148].

Several hypotheses have been advanced incriminating short-acting β_2_-AR agonists as contributing to the second epidemic of asthma deaths that occurred in New Zealand in the late 1970s and early 1980s [146]. One suggested factor was a change in prescribing practices, in that inhaled β_2_-AR agonists, along with sustained-release theophylline preparations, were prescribed in place of inhaled corticosteroids and cromolyn [149], possibly predisposing to fatal cardiac arrhythmias. Another proposed factor was an increased use of β_2_-AR agonists delivered by air-driven home nebulizers for self-treatment of severe asthma. In this case, patients could be exposed to the risk of hypoxic cardiac arrest if large doses of short-acting β_2_-AR agonists were delivered in the face of uncorrected hypoxaemia [150]. Two case-control studies involving asthma deaths in patients aged 5–45 years in New Zealand from 1981 to 1983 [151] and from 1977 to 1981 [152] have led to a particularly contentious hypothesis linking some of the excess in asthma mortality to prescribed fenoterol by metered-dose inhaler. In 1992, Spitzer *et al.* [153], by using health insurance data from Saskatchewan (Canada), found an increased risk of death or near-death from asthma in association with regular use of short acting β_2_-AR agonists. These last included fenoterol (odds ratio, 5.4 per canister), but also salbutamol (odds ratio, 2.4 per canister). The authors concluded that these findings could be due to adverse effects of the β_2_-AR agonists themselves but raised the alternative possibility that their increased use might simply be a marker of more severe disease. A 1 year clinical trial showed increased airway responsiveness and worsened asthma control during regular treatment with fenoterol added to usual therapy compared with short-acting β_2_-AR agonists used only as needed for symptom relief [154]. However, subsequent US and UK trials of regular *versus* as-needed salbutamol did not detect sustained adverse effects on asthma control [116,155]. Nevertheless, in 1997 the British Guidelines on Asthma Management increasingly advocated the use of short-acting β_2_-AR only as needed for symptom relief [156].

### 8.2. Long-Acting β_2_-AR Agonists

The introduction of long-acting β_2_-AR agonists salmeterol [157] and formoterol [158] in the 1990’s was considered a major advance in bronchodilator therapy with evidence that their use led to improved lung function and quality of life [114]. There were also potential safety advantages due to the twice daily, fixed dose usage, which reduced the risk of overuse of β_2_-AR agonist therapy in the situation of severe exacerbations. However, concerns about the possible risks associated with long-acting β_2_-AR agonists therapy were raised soon after their introduction into clinical practice. This was due to the evidence that their regular use had the potential to reduce bronchodilator sensitivity to β_2_-AR agonists [159,160] and could induce tolerance to their bronchoprotective effects [95], which may not be restored by concurrent use of inhaled corticosteroids [161]. It also became apparent that patients using long-acting β_2_-AR agonists may be at risk of severe exacerbations if the symptom control, achieved with long-acting β_2_-AR agonists use, led to a discontinuation of inhaled corticosteroid therapy [162].

There were also concerns about a potential risk of mortality from the Salmeterol Nationwide Surveillance Study [163]. In this UK-based study of >25,000 subjects, there was a non-significant three-fold increased risk of asthma death in subjects on salmeterol compared with regular salbutamol. This led to the US-based Salmeterol Multicenter Asthma Research Trial, a placebo-controlled study of the safety of salmeterol in adults with unstable asthma [164]. The trial was stopped after *an interim* analysis showing a statistically significant, four-fold increase in asthma mortality with salmeterol. By contrast, in a large UK-based case-control study there was no positive association between long-acting β_2_-AR agonists therapy and asthma death [165]. However, during the period of this study, ~95% of UK asthma patients receiving long-acting β_2_-AR agonists therapy were co-prescribed inhaled corticosteroid [166]. Due to conflicting evidence, the Food and Drug Administration (FDA) confirmed the availability of both salmeterol and formoterol, but required black-box warnings on their product labels [167].

Formoterol fumarate is currently the most widely used long-acting β_2_-AR worldwide. Three placebo-controlled trials showed that the higher dose (48 µg/day) of formoterol tended to be associated with more serious asthma exacerbations than a lower one [168]. However, a large phase IV trial found all doses of formoterol to be associated with fewer exacerbations than placebo, with no indication of any dose-response relationship [169]. 

Recently, Sears *et al.* [170] have reported a comprehensive meta-analysis of safety data (n = 68,004 patients) obtained in completed trials. When comparing all formoterol-exposed patients to all patients not exposed to long-acting β_2_-AR agonists in the whole data set, no increased risks of cardiac-related deaths or cardiac-related non-fatal serious adverse events were observed. Similarly, when examined by inhaled corticosteroids use there was no increased risk of cardiac serious adverse events with regimens of formoterol with inhaled corticosteroids *vs.* inhaled corticosteroids without long-acting β_2_-AR agonists. Noteworthy, there was a significant reduction in asthma-related serious adverse events with formoterol (>90% of which were hospitalizations) speaking against a relationship between formoterol and increased asthma mortality. The main limitation of the study by Sears *et al.* [170] was the lack of sufficient power to form a definitive conclusion regarding the risk of death for patients treated with formoterol. 

## 9. Future Prospects and Conclusions

A variety of β_2_-AR agonists with longer half-lives are currently undergoing development, with the hope of achieving once-daily dosing. Among them, the development of indacaterol is very advanced and it is likely that this drug will be launched by the end of 2010 [171,172]. Indacaterol provided effective and sustained 24 h bronchodilator effect, with a rapid onset of action (<5 min) and a good tolerability and safety profile in asthma control [173].

Numerous studies have shown the benefit of adding a long-acting β_2_-AR instead of doubling or increasing the dose of inhaled corticosteroids [136,174,175] and the greater benefits to most outcomes of adding a long-acting β_2_-AR compared with adding a leukotriene antagonist [176]. However, both the International Asthma Treatment Guidelines [5,6] and the FDA [177] now emphasize that long-acting β_2_-AR agonists should not be used as monotherapy in asthma but always together with an inhaled corticosteroid. 

In conclusion, either as rescue medications and therapy added to or combined with inhaled corticosteroids, short- and long-acting β_2_-AR agonists have proved effective in achieving a good control of asthma. In particular, they are essential in reducing daytime and especially nighttime symptoms, improving lung function, reducing the risk of exacerbations, and minimizing the required dose of inhaled corticosteroids.
